# Survival of quick problem solver!

**DOI:** 10.1093/emph/eoae028

**Published:** 2024-10-24

**Authors:** Mesut Tez

**Affiliations:** Department of Surgery, University of Health Sciences, Ankara City Hospital, Cankaya, Ankara, Turkey

To the Editor,

I am writing in response to the article ‘A new perspective on tumor progression: Evolution via selection for function’ by Thomas et al., recently published in Evol Med Public Health. The authors propose a paradigm shift in understanding tumor progression, suggesting that cancer cells evolve based on selective pressures for functional traits within group phenotypic compositions (GPCs). This functional selection, they argue, stands apart from classical Darwinian natural selection based on reproductive fitness. Instead, it illustrates how adaptability and problem-solving play a critical role in the progression of cancer, providing a new lens through which to view evolutionary strategies in oncology.

In my view, survival in hostile environments depends not merely on genetic fitness but on an entity’s problem-solving abilities. This idea is reflected in Thomas et al’.s analysis of tumor microenvironments, where cancer cells enhance their adaptability through functional traits that support their collective resilience. This concept suggests that tumor progression may be less about individual cell survival and more about the tumor’s overall ability to adapt and problem-solve under environmental pressures, a view consistent with Sir Richard Peto’s black box epidemiology, which emphasizes correlation and functional traits over mechanistic details in understanding cancer risk and progression [1, 2].

Thomas et al.’s exploration of GPC evolution reveals a distinct selection mechanism that prioritizes collective adaptability rather than individual fitness. This idea aligns closely with Stuart Kauffman’s autocatalytic model, which demonstrates that self-organizing, interconnected networks stabilize based on functionality within selective environments. Within the tumor ecosystem, cancer cells likewise evolve redundant pathways and cooperative functions to increase their survivability. Here, as in other complex systems, the focus shifts to ‘the quick problem-solvers’—the cancer cells that foster functional traits conferring resilience to the whole cell population, especially within a chaotic and hostile microenvironment [3].

Broadening our understanding of cancer through this framework of functional selection has significant therapeutic implications. Recognizing that tumor survival hinges on collective problem-solving and functional redundancy, we might better target the adaptive mechanisms that enable tumors to thrive. By disrupting GPC functions critical to a tumor’s resilience, we may reduce its adaptability and effectiveness in resisting treatment. This approach moves beyond traditional Darwinian competition to address the tumor’s cooperative, function-based survival strategies.

In conclusion, the concept of ‘survival of the quick problem-solvers’ provides an evolved understanding of tumorigenesis, where function and adaptability—rather than strict genetic fitness—drive survival. I commend Thomas et al. for offering a thought-provoking perspective that invites us to rethink evolutionary processes in cancer progression.
